# Altered Brain Structure and Functional Connectivity Associated with Pubertal Hormones in Girls with Precocious Puberty

**DOI:** 10.1155/2019/1465632

**Published:** 2019-12-24

**Authors:** Tao Chen, Yi Lu, Yu Wang, Anna Guo, Xiaoling Xie, Yuchuan Fu, Bangli Shen, Wenxiao Lin, Di Yang, Lu Zhou, Xiaozheng Liu, Peining Liu, Zhihan Yan

**Affiliations:** ^1^Department of Radiology, The Second Affiliated Hospital and Yuying Children's Hospital of Wenzhou Medical University, Wenzhou 325027, China; ^2^China-USA Neuroimaging Research Institute, Radiology Department of the Second Affiliated Hospital and Yuying Children's Hospital, Wenzhou Medical University, 325027 Wenzhou, Zhejiang, China; ^3^Children's Department of Healthcare, The Second Affiliated Hospital and Yuying Children's Hospital, Wenzhou Medical University, 325027 Wenzhou, Zhejiang, China

## Abstract

Pubertal hormones play an important role in brain and psychosocial development. However, the role of abnormal HPG axis states in altering brain function and structure remains unclear. The present study is aimed at determining whether there were significant differences in gray matter volume (GMV) and resting state (RS) functional connectivity (FC) patterns in girls with idiopathic central precocious puberty (CPP) and peripheral precocious puberty (PPP). We further explored the correlation between these differences and serum pubertal hormone levels. To assess this, we recruited 29 idiopathic CPP girls and 38 age-matched PPP girls. A gonadotropin-releasing hormone (GnRH) stimulation test was performed, and pubertal hormone levels (including luteinizing hormone (LH), follicle-stimulating hormone (FSH), estradiol (E2), prolactin, and cortisol) were assessed. All subjects underwent multimodal magnetic resonance imaging of brain structure and function. Voxel-based morphometry (VBM) analysis was paired with seed-to-voxel whole-brain RS-FC analysis to calculate the GMV and RS-FC in idiopathic CPP and PPP girls. Correlation analyses were used to assess the effects of pubertal hormones on brain regions with structural and functional differences between the groups. We found that girls with CPP exhibited decreased GMV in the left insula and left fusiform gyrus, while connectivity between the left and right insula and the right middle frontal gyrus (MFG), as well as the left fusiform gyrus and right amygdala, was reduced in girls with CPP. Furthermore, the GMV of the left insula and peak FSH levels were negatively correlated while higher basal and peak E2 levels were associated with increased bilateral insula RS-FC. These findings suggest that premature activation of the HPG axis and pubertal hormone fluctuations alter brain structure and function involved in the cognitive and emotional process in early childhood. These findings provide vital insights into the early pathophysiology of idiopathic CPP.

## 1. Introduction

Central precocious puberty (CPP) is caused by the early activation of the hypothalamic-pituitary-gonadal (HPG) axis, which is characterized by the onset of secondary sexual characteristics before 8 years of age in girls or 9 years of age in boys. It is much more common in girls than in boys and is usually idiopathic [1]. CPP can cause a range of physical symptoms (early menarche and short adult stature because of the premature closure of growth plates [1]) and numerous additional mental health problems such as anxiety, aggression, deviant behaviors, irritability, self-injurious behaviors, and substance misuse [2–5]. Moreover, these adverse psychosocial behaviors can persist into adulthood [6].

CPP may be pathophysiologically considered an HPG axis disorder, characterized by a dramatic increase in luteinizing hormone (LH), follicle-stimulating hormone (FSH), and sex hormone (androgens and estrogens) levels at an unusually early age. At present, peak serum LH level exceeding 5 IU/L after stimulation by gonadotropin-releasing hormone (GnRH) or a GnRH agonist is considered to be the diagnostic gold standard for CPP. Previous neuroimaging studies have found aberrant brain structure and function, as well as impaired cognitive functioning, in other HPG axis-related disorders, such as familial male precocious puberty (FMPP) [7, 8], polycystic ovary syndrome [9], and Kallmann syndrome [10, 11]. For instance, in FMPP, another subtype of precocious puberty involving excess androgen production; increased parahippocampal, fusiform gyri, and putamen volumes; and aberrant hippocampal activation in response to fearful faces has been noted [7, 8]. Given that puberty is a sensitive time for brain development [12], which features dramatic fluctuations in sex hormone levels, the question of whether early activation of the HPG axis and pubertal hormone fluctuations affects brain development and cognitive in early childhood arises. Answering this question is critical to improving our understanding of the neuroendocrine basis of the psychological features of precocious puberty.

Estradiol (E2) has structural and functional effects on the central nervous system via its mediation of downstream neuroendocrine processes. E2 regulates neurogenesis, inflammatory processes, and neuronal oxidative stress process, as well as cognitive and affective processes [13–16]. Both in mice and in humans, estrogen deficiency is associated with cognitive impairment and anxiety- and despair-like behaviors [17, 18], which are mitigated by exogenous estrogen [19, 20]. Multiple brain networks critical to cognitive and affective processing, including the default mode network, salience network, and the frontoparietal network, are thought to be mediated by estradiol. In social emotion task functional MRI (fMRI) studies, estradiol concentrations positively correlate with activity in part of the mentalizing network (comprised of the anterior temporal cortex, the posterior superior temporal sulcus, and right temporoparietal junction) [21, 22]. In addition, gonadal hormones are associated with activation of reward-related brain regions in adolescence (e.g., the striatum and nucleus accumbens) [23, 24] and the salience network, which includes the anterior insula and anterior cingulate cortex [25, 26].

In addition to endocrine-mediated changes in brain networks, neurodegeneration is also affected by sex hormones. Several brain structures including the hippocampus and hypothalamus with high concentrations of LH receptors are critical for cognitive processing [27]. Human studies of Alzheimer's disease (AD) have reported that increased LH concentrations may lead to the progression of AD, including neurodegenerative, behavioral, and cognitive decline [28]. However, there are few human studies on the effects of LH and FSH on brain development. In pubertal girls, LH levels are associated with increased white matter (e.g., the cingulum, middle temporal gyrus, and splenium of the corpus callosum) [29]. A longitudinal study of twins from ages 9 until 12 found a positive association between FSH concentrations and the volumes of the left hippocampus, left frontal areas, right cerebellum, and left anterior cingulate and precuneus [30].

Structural MRI and task-based fMRI studies have highlighted the influence of the HPG axis and pubertal hormones on brain development, especially in regions critical to emotion and cognition. The influence of pubertal hormones on resting state (RS) functional connectivity (FC) may further reflect intrinsic brain activation patterns regulated by hormones without a task-related bias. Given this, a multimodal imaging study might provide a more accurate description of the structural and functional changes that occur with HPG dysregulation. To the best of our knowledge, there are no existing multimodal studies that combine structural MRI and resting state fMRI in CPP, making the present study highly unique and innovative.

To examine the effects of premature HPG axis activation on brain structure and function, we investigated structural brain differences in GMV using voxel-based morphometry (VBM) and corresponding abnormalities in resting state (RS) functional connectivity (FC) in girls with idiopathic CPP and age-matched girls with peripheral precocious puberty (PPP). In addition, we examined whether these alterations in brain morphology and RS-FC were associated with pubertal hormone levels. In this exploratory study, we postulated that the early initiation of the HPG axis affects brain structure and multiple brain regions, demonstrating that HPG axis-related volume differences may also indicate RS-FC differences. In addition, pubertal hormones play an important role in these regions. While experimental hormone tests in children are ethically prohibited, the GnRH stimulation test is necessary for the accurate clinical diagnosis of precocious puberty. Thus, idiopathic CPP may be an ideal endocrinological model for HPG axis-related study. We identified girls with PPP, which is independent of the HPG axis [1], as optimal “controls,” thereby controlling for the effect of early development of secondary sexual characteristics [31] and isolating the effects of premature activation on the HPG axis.

## 2. Materials and Methods

### 2.1. Participants

A total of 67 right-handed girls (ages at 6-8) were enrolled from the Child Healthcare Department of the Second Affiliated Hospital of Wenzhou Medical University; 29 girls were diagnosed with idiopathic CPP and 38 girls with PPP. All assessments were performed prior to the study subjects receiving GnRH analog treatment. The reasons for the visit included puberty development examinations and complaint of breast development and/or pain accompanied with or without genital and/or axillary hair development. The majority of these complaints were filed by the patients' parents or guardians. Duration of illness was defined as the number of months between the time of scan and the initial time of secondary sexual characteristic development. Left hand and wrist radiographs were used to evaluate bone maturation. The pubertal stage (Tanner method) of all participants was assessed by both inspection and palpation by one pediatric endocrinologist. The gold standard evaluation method of measuring gonadotropin levels by a GnRH stimulation testing was employed here.

Idiopathic CPP was diagnosed in girls for whom bone age was at least one year older than their chronologic age, Tanner stage ≥ 2 breast development, BMI ranges between 25 and 85% according to their age and sex, normal brain and pituitary MRI, and a peak LH level ≥ 5 IU/L in the GnRH stimulation test. A cut-off of ≥5 IU/L was chosen to differentiate clearly between the study groups (idiopathic CPP and PPP). PPP was diagnosed in girls for whom bone age was at least one year older than their chronologic age, Tanner stage ≥ 2 breast development, BMI ranges between 25 and 85% according to their age and sex, and a peak LH level less than 5 IU/L in the GnRH stimulation test. Exclusion criteria for both groups included the following: (1) premature birth, (2) a history of menstruation, (3) precocious puberty with central nervous system lesions or congenital causes, (4) a history of psychiatric illness (e.g., depression, schizophrenia, attention deficit hyperactivity disorder (ADHD), and conduct disorder (CD)), (5) long-term hormonal treatment, and (6) contraindication to MRI.

All participants underwent thyroid-stimulating hormone (TSH), total T3, total T4, and cortisol profile examination (fasting blood samples at 8:00-8.30 am) to exclude thyroid and adrenal disorders. Pelvic ultrasound and pituitary MRI scans were performed on all girls to exclude ovarian and other pituitary-related disorders.

As human hormone experiments are ethically prohibited, children with PPP were used as controls, controlling for the influence of secondary sexual characteristics on our outcomes. The present study was approved by the Ethics Committee of the Second Affiliated Hospital of Wenzhou Medical University. Informed written consent was obtained from the appointed proxies of all patients and guardians. Additionally, all work was conducted in accordance with the Declaration of Helsinki (1964).

### 2.2. Cognitive and Behavioral Assessment

Full-scale IQ was assessed using the Wechsler Intelligence Scale for Children-Chinese Revised (WISC-CR). Behavior was assessed via the parental report using the child behavior checklist (CBCL) [32]. The Hamilton Anxiety Rating Scale (HAMA) was used to evaluate the severity of anxiety.

### 2.3. Hormonal Assays and GnRH Stimulation Testing

The GnRH stimulation tests were performed at approximately 8:00 am in all patients. Serum LH and FSH levels were measured at 0, 30, and 60 minutes and E2 was measured at 0 and 30 minutes after injected with gonadorelin (2.5 *μ*g/kg, maximum dose 100 *μ*g). Measured maximum levels of serum LH, FSH, and E2 were regarded as peak LH, FSH, and E2 levels, respectively. Hormone levels measured at 0 min were treated as baselines. Blood samples were measured using electrochemiluminescence immunoassay. Hormone concentrations were expressed according to minimum detection values (assay sensitivity = 0.2 IU/L). A peak LH level after GnRH stimulation ≥ 5 IU/L indicated HPG axis initiation [33]. Previously established criteria were used to distinguish central precocious puberty (characterized by sustained HPG axis activation) from nonprogressive precocious puberty (in which the HPG axis is not activated) [1]. Given that the distribution of our hormonal data was negatively skewed (Kolmogorov-Smirnov test, *P* < .05), data were log transformed for subsequent correlation analyses.

### 2.4. MRI Acquisition

Prior to GnRH stimulation testing, participants underwent MRI scanning. Structural and functional MRIs were performed with a 3.0 T GE Healthcare-Signa HDxt 3T MRI scanner (General Electric, Milwaukee) using an eight-channel phase array head coil. We used noise-reducing headphones and sponge pads to minimize head movement. High-resolution 3D structural imaging data was collected using a whole-brain spoiled gradient echo sequence (repetition time (TR) = 8.88 ms; echo time (TE) = 4.02 ms; inversion time = 900 ms; flip angle (FA) = 15°; matrix size = 256 × 256; field of view (FOV) = 256 × 256 mm; and slice thickness = 1 mm, 160 slices). Participants were instructed to lie quietly and keep their eyes closed during the resting state functional scan. Images were acquired with a gradient echo-planar imaging sequence (repetition time (TR) = 2500 ms, echo time (TE) = 40 ms, flip angle (FA) = 90, slice thickness = 4 mm, slice gap = 4 mm, matrix size = 64 × 64, field of view (FOV) = 256 × 256, 34 slices).

### 2.5. Voxel-Based Morphometry Analyses

T1-weighted images were postprocessed using DARTEL-based VBM conducted using SPM8 (https://www.fil.ion.ucl.ac.uk/spm). Prior to data processing, images from each female participant were aligned to an anterior-posterior commissure line. After correcting for the field nonuniformity bias, MRI data were segmented into gray matter (GM), white matter (WM), and cerebrospinal fluid (CSF) and spatially normalized by DARTEL. A mean GM template was created using individual GM images. All images were smoothed using an 8 mm Gaussian kernel at a full width at half maximum (FWHM). Total intracranial volume was measured by calculating the volumes of GM, white matter, and CSF in each participant.

### 2.6. Functional MRI Image Preprocessing

For all resting state fMRI data, all preprocessing was performed using SPM8 (https://www.fil.ion.ucl.ac.uk/spm) and Data Processing Assistant for Resting-State fMRI (http://www.restfmri.net). The first ten frames were discarded to account for any hemodynamic delay. The preprocessing steps comprised a slice-timing correction for interleaved acquisitions, a head motion correction (head motion was <2.5 mm translation in *x*, *y*, or *z* directions or <2.5° of angular rotation along the three axes), spatial registration of high-resolution structural T1 images to each participant, and adjustment of image time series to remove linear drift. Normalized images were then smoothed with a 6 mm FWHM isotropic Gaussian kernel. Several nuisance signals, including the six motion parameters, white matter, and CSF, were regressed for temporal correction. RS images were then bandpass filtered between 0.01 and 0.08 Hz to reduce low-frequency drift and physiological high-frequency respiratory and cardiac noise. Segmentation of the anatomical image into GM, WM, and CSF and normalization of anatomical and functional images to the standard Montreal Neurological Institute (MNI) brain space (voxel size = 3 mm^3^) were then performed.

### 2.7. Seed-Based Functional Connectivity Analyses

Regions showing differences in the VBM analysis were used as seeds. Based on the peak voxel difference of each significantly different region (per the VBM analysis), 6 mm spherical seed regions of interest at each region were used for seed-to-voxel whole-brain analyses. A Pearson correlation analysis was performed between the time course extracted from each of the seeds and the time courses of all voxels in the rest of the brain, resulting in individual FC maps. Each individual subject's FC map was transformed to a *Z* value map via Fisher's transformation for subsequent group level analyses.

### 2.8. Statistical Analyses

In our comparisons of demographic, psychometric, and clinical hormonal measures, a two-sample Student's *t*-test was applied when samples had a standard normal distribution. The Mann-Whitney *U* test was used when samples exhibited a skewed distribution. A two-sample *t*-test was then used to compare GMV between groups, with age as a covariate. Different brain regions were represented by a standardized MNI coordinate system, and GM intensity values for each individual were then extracted for further analyses. Significance was set to an uncorrected *P* < 0.001, with a voxel number > 12, which corresponded to a corrected *P* < 0.05. Voxel-wise two-sample *t*-tests were used to examine differences in RS-FC between CPP patients and PPPs, with age at scan as a covariate. The significance level for each seed was defined at the voxel level by *P* < 0.001. AlphaSim multiple comparison corrections were performed within AFNI software (https://afni.nimh.nih.gov/afni). The voxel-wise threshold was set to *P* < 0.005 and the cluster threshold was set to >25 voxels. Partial correlation analyses were conducted to assess relationships between GM voxel intensity (where there was a significant difference in structural volume between groups) and clinical variables that were different between groups, controlling for participant age at scan. FC values (where there was a significant difference between groups) were extracted for each participant. Similar analyses were performed to determine associations between differences in RS-FC and clinical variables. *P* < 0.05 was considered statistically significant. All statistical analyses were performed using SPSS version 25.

## 3. Results

### 3.1. Clinical Characteristics and Levels of HPG Axis Hormones

Of the 67 female patients enrolled in this study, nine were excluded from all analyses, including six patients with CPP and three with PPP, due to excessive motion during fMRI acquisition. Thus, the final study sample consisted of 25 female participants with idiopathic CPP (7.27 ± 0.77 years) who were age-matched to 33 female participants with PPP (7.12 ± 0.94 years). We found no difference in child behavioral checklist outcomes or clinical characteristics (age, bone age, height, weight, BMI, and duration of illness) between the CPP and PPP samples. Demographic characteristic and psychometric data for the entire patient sample are in Table 1.

There were significant differences in basal LH levels (*P* = 0.019), peak LH levels (*P* = 0.001), basal E2 levels (*P* = 0.007), peak E2 levels (*P* = 0.018), and LH/FSH ratios (*P* = 0.003) between the groups. However, no significant differences were found in basal FSH levels (*P* = 0.264), peak FSH levels (*P* = 0.215), PRL levels (*P* = 0.685), or COR levels (*P* = 0.655) between the two groups. The descriptive statistics for hormone level outcomes are summarized in Table 2.

### 3.2. Gray Matter Volume

**Figure 1:**
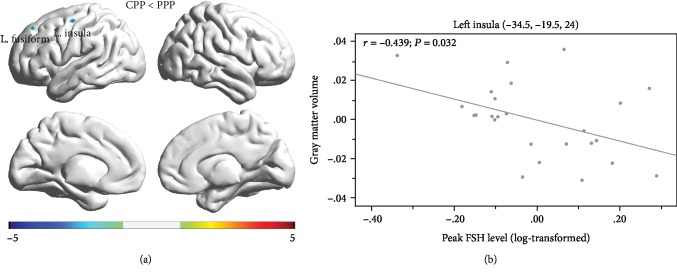
(a) Decreased gray matter density in central precocious puberty (CPP) vs. controls for the left insula and left fusiform overlaid on a 3D-T1 image in MNI space. All results shown are significant at *P* < 0.05 (FDR). (b) Central precocious puberty (CPP) group lower peak FSH level (*r* = −0.439, *P* = 0.032) is associated with increased GMV in the left insula.

Relative to controls, CPP patients showed lower GMV in the left insula and the left fusiform gyrus (see Table 3 for a summary and Figure 1(a) for a visual depiction). Lower GMV in the left insula was associated with increased peak FSH levels (*r* = −0.439, *P* = 0.032) in pediatric patients with CPP (see Figure 1(b)). No correlations were observed with any other HPG axis hormones. Results were corrected for age at scan.

### 3.3. Resting State Functional Connectivity

**Figure 2:**
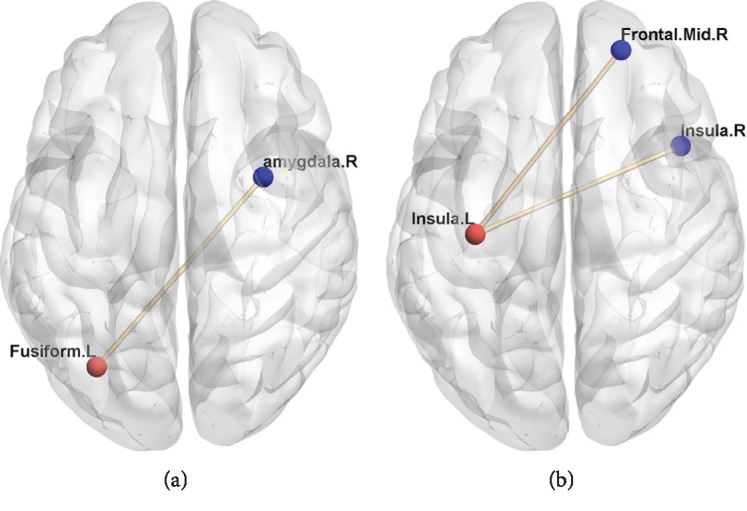
RS-FC differences between CPP and PPP. The figure shows girls with CPP having lower connectivity from the left insula to the right insula and the right middle frontal gyrus and from left fusiform to the right amygdala. All results shown are significant at *P* < 0.005 (AlphaSim). (a) The left fusiform as a seed. (b) The left insula as a seed.

**Figure 3:**
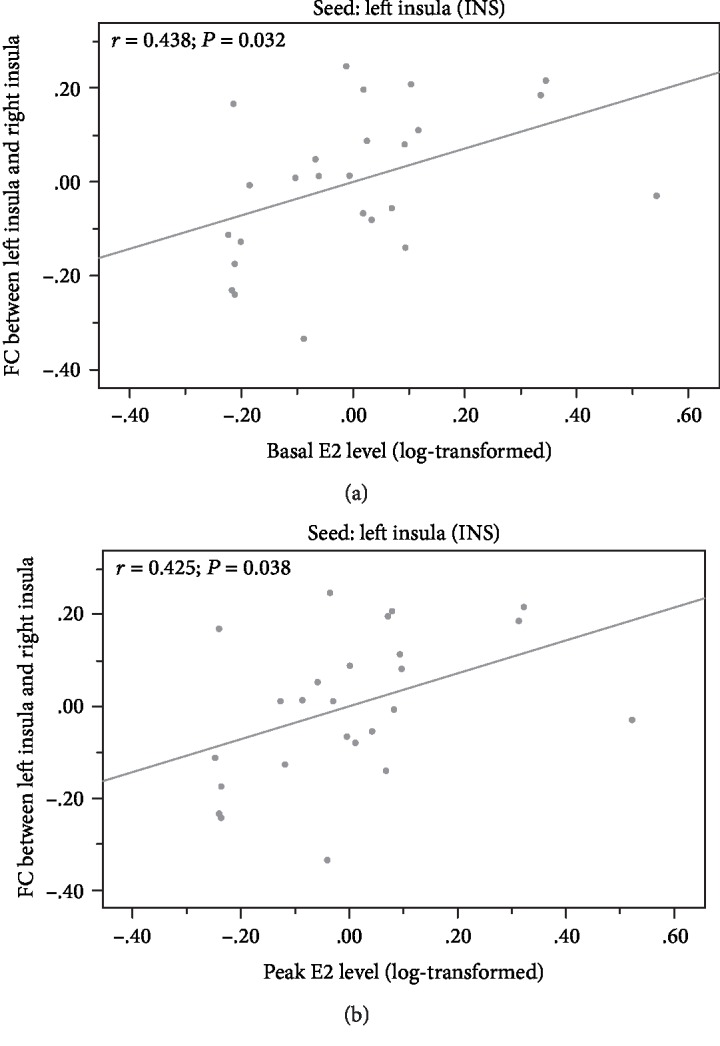
Correlation analysis between FC values and E2 level in the CPP group, stronger FC within the bilateral insula is associated with increased (a) basal and (b) peak E2 level (basal: *r* = 0.438, *P* = 0.032; peak: *r* = 0.425, *P* = 0.038).

The present study revealed regional structural changes and decreased FC in girls with CPP. Patients with CPP demonstrated lower connectivity than those with PPP from the left insula to the right insula and the right middle frontal gyrus (MFG), as well as from the left fusiform gyrus to the right amygdala (see Table 4 for a summary and Figure 2 for a visual depiction). This increased FC between the left insula and the right insula was further correlated with increased basal E2 levels (see Figure 3(a)) (*r* = 0.438, *P* = 0.032) and increased peak E2 levels (see Figure 3(b)) (*r* = 0.425, *P* = 0.038) in the CPP group.

## 4. Discussion

Our multimodal imaging investigation revealed alterations in brain structure and functional connectivity in girls with CPP. We found decreased GMV in the left insula and left fusiform gyrus, areas involved in salience networks and facial recognition, respectively. Girls with CPP also exhibited decreased RS-FC within the sensorimotor network (insula-L and insula-R), between the fusiform and salience (amygdala) network, and between the salience (insula-L) and executive attention (MFG) networks. These findings indicate an association between HPG axis state and RS-FC in the cortical and subcortical regions involved in cognitive and emotional processing. Furthermore, in girls with CPP only, peak FSH levels were negatively correlated with GMV within the left insula. In addition, basal and peak E2 levels were positively correlated with FC between the left and right insulae. These findings suggest that communication in the salience network may be modulated by FSH and E2 concentrations in CPP.

Girls with CPP exhibit decreased GMV in the insula and fusiform gyrus, regions which are implicated in affective, cognitive, and regulatory functions [34], as well as facial recognition [35]. Specifically, the insula is critical for social [36, 37] and empathic processes [38, 39], as well as interoceptive accuracy and subjective evaluation scores of visceral consciousness [40]. This supports the existence of a strong role for the insula in perception of internal bodily states and emotional experiences [34]. Girls with CPP are prone to social and emotional adjustment problems. A hypothesis proposed by Brooks-Gunn and colleagues suggest that hormone fluctuations may have a biological effect on individual susceptibility to adjustment problems, rather than via changed secondary sexual characteristics [41]. Our results provide evidence for a direct biological effect of hormone changes due to the early initiation of the HPG axis on social and emotional processing, which might be independent of pubertal stage. Furthermore, the close association between early initiation of the HPG axis and the insula might serve as the neuroendocrine basis of the clinical features of CPP.

Despite the lack of information on brain development in CPP, some studies have assessed the influence of HPG axis fluctuations on fusiform structure and function. GMV abnormalities in the fusiform gyrus have been reported in FMPP [7]. Furthermore, FMPP individuals exhibit faster processing of fearful faces during perception of a threat than controls [8]. Stronger brain responses to faces in the right fusiform face area were also observed in women taking oral hormonal contraceptives versus not and in women during midcycle compared with menstruating women [42].

Girls with CPP also exhibited decreased RS-FC between the insula and the MFG, suggesting lower integration between the salience network and the executive attention network. Decreased connectivity between the bilateral insulae further suggests weaker intrinsic connectivity in the salience network in CPP patients. The MFG serves as an executive attention network hub and is involved in high-order cognitive and emotional behavior regulation [43, 44]. Aron et al. found that the severity of damage in the MFG is closely related to the degree of top-down control [45]. Furthermore, girls with CPP also exhibited lower RS-FC between the fusiform gyrus and the amygdala, suggesting lower integration between social processing and the salience network.

The amygdala, a critical limbic structure, also plays a role in emotion processing and introspection. Attenuated functional activity in the amygdala is seen in early childhood onset depression [46] and in conduct disorder [47]. Decreased corticolimbic FC, as was found in the MFG, may indicate decreased top-down control over emotional regulation. It is worth noting that insula-based abnormal connectivity has been reported in several psychiatric diseases (e.g., depression, schizophrenia, ADHD, and CD) which often first present during puberty [44–47].

Based on the results discussed above, our negative cognitive and behavioral assessment findings in the two groups included here should be interpreted with caution. Our results suggest that, during early childhood (ages at 6-8), premature activation of the HPG axis does not have large or significant adverse effects on cognitive and affective development. However, our findings cannot rule out the possibility that significant behavioral abnormalities may occur after long-term exposure to a prematurely activated HPG axis. Great attention should be paid to the mental health of CPP patients. The present study is the first to test for an association between premature activation of the HPG axis and cognitive and emotional neural network dysregulation in early childhood and as such serves as only a start for the field. Future work including a larger sample size and longer duration of illness will be crucial for clarifying these effects.

In the present study, we found that FSH levels were positively associated with GMV in the insula. Few studies have investigated the role of FSH in human brain development, though previous animal studies have demonstrated that transcription products FSH protein can be identified in the central nervous system [48]. FSH and its receptor are present in the brain, specifically in the cerebellar cortex [49] and hippocampus [50] of the rat. In humans, Lu et al. [51] revealed that high serum FSH levels were positively associated with GMV in the right pallidum in perimenopausal women. A significant positive association between FSH levels and gray matter density in the hippocampus, frontal gyrus, cerebellum, anterior cingulate, and precuneus has also been observed in typically developing 9-year-old children [30]. Despite this, however, no associations between FSH and the brain volume of adolescents aged 9-15 have been found [29, 52]. The present study is thus consistent with previous MRI reports revealing that FSH may play a critical role in brain development. It should further be noted that this finding only appeared in group with the activation of HPG, although no difference in FSH levels between the two groups was found. One possible explanation is that FSH has organizational and activating effects on the brain only in the specific HPG axis state. As the upstream hormone of FSH, GnRH receptor has been found to colocalize with FSH in some neurons of the central nervous system, which indicated that the function of FSH in the central nervous system may be regulated by GnRH [49, 50]. At the activated status of HPG axis, the pulsatile release of GnRH may thus stimulate the function of FSH in brain development. In other words, the effect of FSH on brain development may become significant only after the HPG axis is activated.

E2 levels were positively correlated with RS-FC between the bilateral insular cortices (insula-L, insula-R), critical elements of the salience network, in the present study. Previous reports have found that estradiol mediates ventral medial prefrontal cortex and amygdala reactivity and emotional processing (e.g., fear extinction) [53]. A social emotion task fMRI study in girls (aged 11.1-13.7 years) similarly found that estradiol levels were positively correlated with activity in the social emotion processing-related network [21]. The results of the present study are consistent with previous fMRI findings in healthy young females [54], revealing that the FC of multiple brain networks implicated in emotional processes may be partly modulated by E2 levels.

Finally, despite its contributions to the field, the present study has some limitations which warrant consideration. First, it utilized a cross-sectional design that rendered us unable to investigate any causal relationship between premature activation of the HPG axis and developmental brain changes. Second, to gain a better understanding of the pathophysiology of CPP during early childhood specifically, we excluded children older than 8 years or those who had experienced menarche. Our findings may thus not generalize to children with a longer course of CPP or more significant behavioral problems. Third, the sample size used in the present study was relatively small, and future, larger studies are required to further substantiate our findings. Despite these limitations, however, the present study suggests that brain development in CPP leads to structural and functional differences in early childhood. Future studies which focus on CPP patients that already manifested psychological abnormality are needed to investigate the relationship between the RS-FC within the above networks and the clinical outcomes. It would be necessary to assess whether FC of these networks has any predictive power, potentially suggesting that children with CPP are at risk for psychiatric disorders in the future.

## 5. Conclusions

The present study examined the differences in brain structure and function between girls with a diagnosis of CPP and PPP. Decreased GM in regions involved in cognition and emotion was associated with FSH levels. In addition, dysregulated RS-FC within insula, a region involved in the salience network, was also related to E2 levels in the CPP group. This suggests that decreased gray matter volume and functional connectivity in regions involved in cognitive and affective processing and facial recognition may be mediated by premature activation of the HPG axis in childhood. The present study deepens the present understanding of the neuroendocrine basis of early CPP. Further research is required to investigate the relationship between long-term HPG axis dysfunction and susceptibility to psychiatric disorders.

## Figures and Tables

**Table 1:** Clinical characteristics of participants.

Variables	CPP group, *n* = 26 Mean ± SD	PPP group, *n* = 33 Mean ± SD	*P* value
Age (year)	7.27 ± 0.77	7.12 ± 0.94	0.499
Bone age (year)	8.69 ± 1.35	8.03 ± 1.21	0.055
Weight (kg)	26.13 ± 4.34	26.48 ± 4.72	0.769
Height (cm)	126.30 ± 6.22	125.92 ± 7.02	0.835
BMI (kg/m^2^)	16.37 ± 2.46	16.60 ± 1.86	0.683
Duration of illness	6.66 ± 3.74	5.84 ± 2.40	0.340
IQ	108.17 ± 17.30	106.25 ± 13.96	0.232
CBCL			
Total scores	9.58 ± 10.28	9.86 ± 10.09	0.821
Activities	5.73 ± 2.76	6.05 ± 2.42	0.806
Social contact	6.80 ± 2.43	6.48 ± 2.89	0.736
Learning	5.01 ± 0.73	5.37 ± 0.82	0.795

CPP: central precocious puberty; PPP: peripheral precocious puberty; CBCL: child behavior checklist.

**Table 2:** HPG axis hormone levels of the two groups.

Subject	CPP group, *n* = 26	PPP group, *n* = 33	*P* value
Basal LH (IU/L)	0.23 ± 0.23	0.11 ± 0.08	0.019
Peak LH (IU/L)	9.72 ± 8.54	3.30 ± 1.10	0.001
Basal FSH (IU/L)	2.98 ± 1.67	2.55 ± 1.03	0.264
Peak FSH (IU/L)	17.04 ± 6.32	15.09 ± 5.46	0.215
Basal LH/FSH	0.66 ± 0.63	0.24 ± 0.10	0.003
Basal E2 (IU/L)	36.69 ± 21.49	24.27 ± 12.33	0.007
Peak E2 (IU/L)	38.37 ± 21.00	27.25 ± 13.58	0.018
PRL (IU/L)	12.30 ± 6.82	11.59 ± 6.31	0.685
COR (IU/L)	12.35 ± 5.17	11.67 ± 6.10	0.655

LH: luteinizing hormone; FSH: follicle-stimulating hormone; E2: estradiol; PRL: prolactin; COR: cortisol.

**Table 3:** Regions of group differences in gray matter volume.

Region	Brodmann area	*T* value	Cluster size (mm^3^)	MNI coordinates		
				*x*	*y*	*z*
L insula	13	3.75	13	-34.5	-19.5	24
L fusiform	19	3.84	21	-31.5	-70.5	-9

MNI: Montreal Neurological Institute.

**Table 4:** Regions of group differences for seed-based functional connectivity analysis.

Seed	Region	Side	Ke	*t* value	MNI coordinates		
					*x*	*y*	*z*
	Contrast: CPP < PPP						
L insula	Insula	R	37	-4.25	45	15	-9
	MFG	R	38	-3.94	22	52	26
L fusiform	Amygdala	R	26	-4.26	33	2	-21

CPP: central precocious puberty; PPP: peripheral precocious puberty; R: right; L: left; MFG: middle frontal gyrus; MNI: Montreal Neurological Institute.

## Data Availability

The experimental data used to support the findings of this study are available from the corresponding author upon request.

## Abbreviations

CPP: Central precocious puberty
PPP: Peripheral precocious puberty
HPG: Hypothalamic-pituitary-gonadal
GnRH: Gonadotropin-releasing hormone
LH: Luteinizing hormone
FSH: Follicle-stimulating hormone
E2: Estradiol
RS: Resting state
FC: Functional connectivity
GMV: Gray matter volume
VBM: Voxel-based morphometry
MFG: Middle frontal gyrus
CBCL: Child behavior checklist.
